# An analysis of analgesia and opioid prescribing for veterans after thoracic surgery

**DOI:** 10.1038/s41598-020-68303-9

**Published:** 2020-07-09

**Authors:** Matthew J. Pommerening, Aaron Landau, Katherine Hrebinko, James D. Luketich, Rajeev Dhupar

**Affiliations:** 1 Department of Cardiothoracic Surgery, University of Pittsburgh School of Medicine, 200 Lothrop Street, Suite C800, Pittsburgh, PA 15213 USA; 2 Surgical Services Division, Veteran’s Affairs Pittsburgh Healthcare System, University Drive C, Pittsburgh, PA 15240 USA

**Keywords:** Drug regulation, Risk factors

## Abstract

The opioid crisis is a public health issue and has been linked to physician overprescribing. Pain management after thoracic surgery is not standardized at many centers, and we hypothesized that excessive narcotics were being dispensed on discharge. As a quality improvement initiative, we sought to understand current prescribing practices to better align the amount of opioids dispensed on discharge to actual patient needs. This was a single-center, retrospective review of patients undergoing thoracic surgery from 7/2015 to 7/2018. Demographics, operative data, perioperative pain medication use, and discharge pain medication prescriptions were analyzed. Opioids were converted to Morphine Milligram Equivalents (MME). Among 124 patients, 103 (83%) received intraoperative nerve blocks and 106 (85.5%) used PCAs. Prescribed MME/day at discharge were significantly higher than MME/day received during hospitalization (Median 30 [IQR 30–45] vs. 15 [IQR 5–24], p < 0.001) and were not associated with receiving a nerve block or PCA. By procedure, prescribed MME/day were significantly higher than inpatient MME/day for wedge resections (p < 0.001), segmentectomies (p = 0.02), lobectomies (p = 0.003), and thymectomies (p = 0.02). Patients are being discharged with significantly more opioids than they are using as inpatients. Education among prescribers and a standardized approach with patient-specific dosing may reduce excessive opioid dispensing.

## Introduction

The opioid crisis is a public health issue that impacts many people in the United States^1,2^. In recent years, opioid abuse has increasingly been attributed to prescription pain medications and physician overprescribing^3^. Its estimated that over a quarter billion opioid prescriptions are written each year in the United States, yielding a potential excess supply of narcotics in the community that may contribute to overdoses, addiction, and availability for illicit use^4–6^.

Postoperative analgesia after thoracic surgery, however, can be particularly challenging. Pain management plays an important role in facilitating early mobilization and preventing respiratory complications. However, the presence of postoperative thoracostomy tubes, sometimes multiple, can result in intercostal nerve pain in addition to the usual incisional pain. Even with multimodality non-narcotic analgesia, the use of narcotics is often an important component of recovery from surgery.

An important facet of opioid stewardship by physicians is to appropriately prescribe narcotics on discharge, thereby reducing the potential for abuse and distribution in the community. As a quality improvement initiative to decrease excessive outpatient narcotics prescriptions after surgery, we first sought to define current prescribing practices to better align the amount of opioids dispensed on discharge to actual patient needs. We hypothesized that excessive narcotics were being dispensed. This is the first part of an opioid stewardship program targeted toward decreasing unneeded narcotics dispensing at the institution.

## Methods

This is a single-center retrospective review of patients undergoing thoracic surgery procedures at VAPHS from 7/2015 to 7/2018. Patients undergoing limited invasive or endoscopic procedures, such as diagnostic thoracentesis, tunneled pleural catheters without VATS, bronchoscopy, and mediastinoscopy were excluded. Additionally, all patients undergoing abdominal and foregut procedures, such as paraesophageal hernia repair and esophagectomy, were excluded. Patients were also excluded if they underwent additional non-thoracic surgery during the same hospitalization or died prior to discharge. Data reviewed included patient demographics, preoperative use of pain medications, operative data, inpatient pain medication use, and pain medications prescribed at discharge. It is routine practice to perform intraoperative intercostal nerve blocks at this institution, therefore none of the patients in the study received epidural anesthesia or topical lidocaine patches during the postoperative period. Opioid-based medications were converted to Morphine Milligram Equivalents (MME) for standardization and comparison using the conversion factors shown in Table 1. Inpatient medications included patient-controlled analgesia (PCA) pumps, oxycodone, oral acetaminophen, and non-steroidal anti-inflammatory medications in the form of either oral ibuprofen or intravenous ketorolac. PCA use was quantified by the number of hours the PCA was active from the time of surgery as well as the total dose of hydromorphone used, converted to MME. MME/day at discharge was calculated using the dose and frequency prescribed. That is, multiplying the milligrams of opioid per dose (converted to Morphine Milligram Equivalents) × the frequency prescribed per day.

**Table 1:** Morphine milligram equivalents (MME) conversion factors.

Drug	Conversion factor
Acetaminophen-codeine	0.15
Hydrocodone-acetaminophen	1.5
Hydromorphone	4
Morphine	1
Oxycodone	1.5
Oxycodone-acetaminophen	1.5
Tramadol	0.1

Continuous data are presented as medians and interquartile ranges (IQR) and categorical data are presented as proportions. Comparisons between groups were tested for statistical significance using Wilcoxon rank sum tests for continuous variables and χ^2^ or Fisher exact tests for categorical comparisons. All statistical analyses were performed using STATA Statistical software (version 13; College Station, TX, USA). This study was approved by the institutional review board of the Veteran’s Affairs Pittsburgh Healthcare System as a Quality Improvement Initiative. All methods were carried out in accordance with relevant guidelines and regulations. Informed consent was waived for this study.

### Conference presentation

Presented at the “2019 Association of VA Surgeons Annual Meeting” in Seattle, WA on April 27–30.

## Results

A total of 418 patients underwent thoracic surgery procedures during the study period. Of these, 72 patients underwent foregut procedures and 222 patients underwent endoscopic or bedside procedures and were excluded. A total of 124 patients meeting study criteria were reviewed. The majority of patients in this veteran population were older, white males most commonly presenting with non-small cell lung cancer or new lung or pleural nodules (Table 2). The majority of patients received lobectomies followed by diagnostic wedge resections or pleural biopsies (Table 3). VATS was the most common approach (84.7%), followed by robotic (8.1%) and thoracotomy (7.2%). Four patients returned to the operating room. The median LOS was 5 days (IQR 3–9). There were no mortalities within 30 days of discharge.

**Table 2:** Demographics for 124 patients undergoing thoracic surgery.

Variable*	N
Age	69(64–73)
Male	117 (95.1)
White race	113 (91.1)
Smoking history	60 (48.4)
Home narcotic use	27 (21.8)
**Diagnosis**	
NSCLC	65 (52.4)
SCC	4 (3.2)
Pulmonary/pleural nodule	26 (21.0)
Lung metastasis	2 (1.6)
Thymic mass	6 (4.8)
Pneumothorax, pleural effusion	17 (13.7)
Other	4 (3.2)

*NSCLC* non-small cell lung cancer, *SCC* small cell carcinoma.

*Data are reported as numbers (percentiles) and medians (interquartile ranges).

**Table 3:** Operative data for 124 patients undergoing thoracic surgery.

Procedure*	
Wedge resection/pleural biopsy	41 (33.1)
Lobectomy	43 (34.7)
Segmentectomy	16 (12.9)
Thymectomy	6 (4.8)
Decortication	6 (4.8)
Thoracotomy	9 (7.3)
Other**	3 (2.4)
Case duration (min)	183 (130–250)
Intraoperative intercostal nerve block	103 (83.1)
PCA	106 (85.5)
PCA duration (h)	34 (21–48)
LOS (days)	5 (3–9)
Return to operating room	4 (3.3)

*PCA* patient-controlled analgesia, *LOS* length of stay.

*Data are reported as numbers (percentiles) and medians (interquartile ranges).

**Diaphragm plication, resection of thoracic esophageal leiomyoma, and thoracic duct ligation.

Twenty-seven patients (21.8%) were taking pain medication preoperatively, and of these, 18 patients were taking opioids (oxycodone, hydrocodone, hydromorphone, codeine, or MS Contin), while the remaining 9 patients were taking the partial opioid agonist, tramadol. The most common reason for use of pain medication preoperatively was back pain or osteoarthritis, however migraines and fibromyalagia were also listed as reasons.

One hundred three patients (83.1%) received intraoperative intercostal nerve blocks, including 22 of the 27 patients taking pain medications preoperatively. Postoperatively, all 124 patients were prescribed a hydromorphone PCA, however 18 did not use it, and 106 (85.5%) patients used the PCA for a median duration of 34 (21–48) h. There were no significant predictors for which patients used the PCA. Non-opioid medications were ordered for 100 (80.6%) patients during the inpatient stay. Of the patients receiving additional non-opioid medications, 72 (58%) patients received oral acetaminophen, 79 (63.7%) received intravenous ketorolac, 17 (13.7%) received oral ibuprofen, and 56 (45%) patients received a combination of one or more of the non-opioid medications. Five patients received between one and five doses of tramadol during hospitalization, one of whom was taking the medication preoperatively for neck and back pain. One patient also received codeine-acetaminophen, also noted to be a home medication. There were otherwise no significant predictors for which patients received multimodal pain regimens. Moreover, use of intercostal nerve blocks (p = 0.65) and non-opioid medications (p = 0.74) was not associated with a significantly lower amount of inpatient opioids.

A total of 102 (82.9%) patients received prescription pain medication at the time of discharge. The remaining patients were not discharged with any pain medication. All prescriptions were for opioid-based medications. There were no prescriptions given for tramadol, acetaminophen, or non-steroidal anti-inflammatory drugs, and no patients were discharged with more than one pain medication. Ninety-six (94%) patients were given prescriptions for oxycodone, which represented the overwhelming majority of the prescriptions for opioid medications given. Three patients received a combination opioid drug containing acetaminophen (two hydrocodone-acetaminophen, one oxycodone-acetaminophen), one of which was taking the medication preoperatively. There were two prescriptions for hydromorphone and one prescription for morphine sulfate, however these were each previous home medications.

Opioids were prescribed in quantities ranging from 10 to 90 tablets with a median quantity of 30 tablets (IQR 20–50) and median supply of 7 days (IQR 5–10). On average, patients were dispensed 33.6 MME/day at discharge, with a range of 0–135 MME/day. The amount of opioids dispensed at discharge was not associated with the procedure performed, including minimally invasive versus thoracotomy, receiving a nerve block, or use of a PCA. Patients taking chronic pain medications preoperatively used significantly more total inpatient MMEs than patients not on preoperative pain medications (median 217 vs. 45, p < 0.001), and were also prescribed significantly higher total MME at discharge (330 vs. 218, p = 0.007).

**Figure 1:**
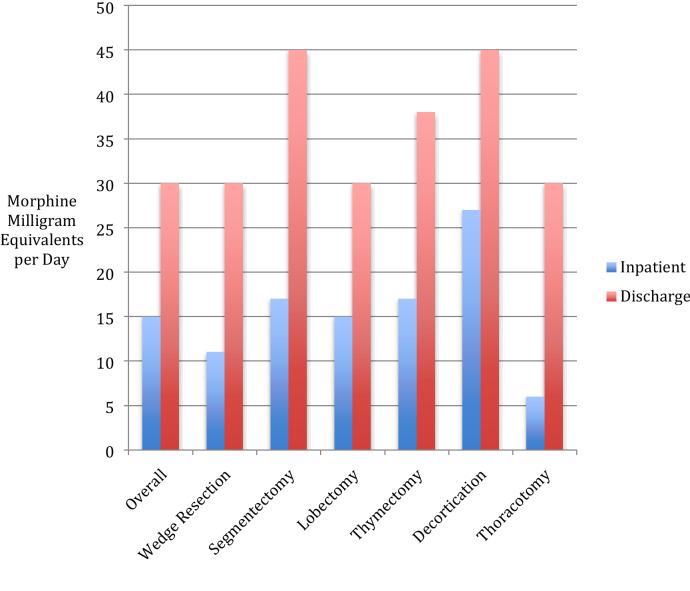
Median inpatient opioid use and prescribed opioids at discharge after thoracic surgery.

Among all patients, significantly more daily MMEs were dispensed at discharge than were used during hospitalization (median 30 [30–45] vs. 15 [5–24], p < 0.001). This was also true by procedure for patients undergoing wedge resections (p < 0.001), segmentectomies (p = 0.02), lobectomies (p = 0.003), and thymectomies (p = 0.02, Fig. 1).

## Discussion

In this study, we aimed to identify and describe current practices of postoperative analgesia after thoracic surgery at our institution. We hypothesized that excessive narcotics were being dispensed when compared with what patients used during hospitalization. We found this to be correct.

The significance of these findings is particularly important in the context of the current opioid crisis and national efforts to reduce prescription opioid misuse and abuse. The fact that patients are being discharged with significantly more opioids than they are using as inpatients raises concern that current practices may be contributing to the excess opioid supply in the community and further supports the implication of physician overprescribing as a major factor in the current opioid problem. There have been programs implemented at many institutions to reduce overall narcotics consumption while improving patient outcomes in thoracic surgery^7,8^. Key elements include factors that help achieve optimal pain control in order to promote early mobilization and reduced hospital length of stay (LOS)^9–11^. However, a multimodal approach to pain management often includes narcotics, and a factor that impacts post-operative addiction is prescribing practices.

The findings from this study were the first part of a multi-step initiative to improve opioid stewardship at our institution. For example, through this study we have identified the lack of prescribing multi-modal pain control in the outpatient setting, despite its use in the immediate postoperative period, as an actionable target for intervention. While this observation is potentially confounded by availability of over-the-counter non-narcotic pain control, there are cost and behavioral incentives to prescribe these medications and they were not prescribed to a single patient during the study time period. We are also implementing a prescriber education program and standardized order sets across multiple disciplines that discourage excessive narcotics dispensing after surgery. The details and outcomes of these programs are the subject of ongoing study by our Opioid Stewardship Committee that has since been created based on the findings of this study. The multidisciplinary committee is comprised of surgeons, internal medicine physicians, and pharmacists.

Through our Committee we have confirmed that interest in changing long standing practices varies across providers and education on the topic is not a high priority without encouragement. Interestingly, the lack of correlation between use of a PCA, nerve block, or multimodal pain regimen with opioids prescribed suggests inpatient factors are not influencing outpatient prescription practices. These findings also suggest there is a significant disconnect between opioid needs and the opioids prescribed at discharge and highlights the need for developing standardized pain medication use assessments for postoperative prescribing, which is being undertaken currently as an institutional program. This approach will focus on more individualized, patient-specific factors including preoperative opioid exposure, procedure type, and inpatient postoperative opioid needs in order to stratify patients into narcotic need categories. Additionally, the prescribing and dispensing of non-narcotic pain medications at discharge will possibly further reduce the need for these higher risk drugs. The goal of this program is to decrease narcotic dispensing by 50–75% after surgery.

It is important to note that the retrospective data reviewed in this study only evaluated the amount of narcotics that were prescribed and does not take into account the patient’s actual usage of the prescribed medication. Although patients were often prescribed more narcotics at discharge than they were using in the hospital, it is unclear how many patients used the prescribed quantities or required additional narcotics and returned to the hospital or clinic sooner than follow up or sought additional prescriptions from other providers. However previous studies have demonstrated similar overprescribing patterns, noting as low as 27% use of the total amount of opioid prescribed^12^.

## Conclusion

Opioids remain an important component of postoperative analgesic therapy after thoracic surgery at our institution and may be overprescribed at the time of discharge. A standardized approach to pain management using multimodal therapies, patient-specific dosing, and provider education may reduce excessive opioid dispensing.
